# Complex atypical caregiving: navigating the margins of unique caregiver roles and burdens

**DOI:** 10.3389/fpsyg.2026.1819388

**Published:** 2026-08-24

**Authors:** Krystal D. Mize, David Hage, Solange Gonzalez, Brenda Hage

**Affiliations:** 1 Department of Psychology, Shady Rest Institute on Positive Aging, Florida Gulf Coast University, Fort Myers, FL, United States; 2 Department of Social Work, Shady Rest Institute on Positive Aging, Florida Gulf Coast University, Fort Myers, FL, United States; 3 Department of Psychology, Florida Gulf Coast University, Fort Myers, FL, United States; 4 School of Nursing, Florida Gulf Coast University, Fort Myers, FL, United States

**Keywords:** caregiver burden, caregiver types, caregiving, complex atypical caregiving, dementia

## Abstract

The incidence and prevalence of Alzheimer’s disease and related dementias (ADRDs) significantly impact care recipients, caregivers, and broader society. Most caregivers of ADRD care recipients are family members. These informal caregivers often experience health, emotional, and practical difficulties in their roles as informal caregivers of loved ones with ADRDs. Most family caregivers are adult children or spouses of ADRD care recipients. Complex atypical caregiver roles may include role reversal, unique caregiving dynamics, or multiple roles. For example, these roles may include a minor child taking care of their parent with a young onset condition, someone with an ADRD diagnosis also taking care of their loved one with a similar condition, or a parent taking care of their child with a neurodegenerative condition that would more typically involve the child taking care of the parent. This perspective format manuscript will discuss unique atypical caregiver roles with emphasis on common challenges experienced by this unique caregiver group and potential for resilience. Recommendations will include exploration of opportunities to increase attention to the topic of atypical caregiver roles, improve clinical practice strategies, and promote additional research focused on complex atypical caregivers and the individuals with ADRDs.

## Introduction incidence/prevalence of ADRDs

The incidence and prevalence of Alzheimer’s disease and related dementias (ADRDs) have amassed significant scientific and public attention related to the increasing rates of these conditions, particularly among aging populations. According to long referenced data from the Framingham study, 20% of women and 10% of men are likely to develop dementia at age 45 with risks increasing as people exceed age 65 (Chene et al., 2015). More recent modeling by Fang et al. (2025) estimates the lifetime risk of developing dementia at one in every 2.4 (42%) people after age 55 with a 95% confidence interval. Populations with an even higher lifetime risk ranging from 45 to 60% include women, Black adults, and people carrying the *APOE* ε4 gene variant. Chronological age is positively associated with the development of ADRD, but young onset dementias (YOD) under the age of 65 also remain an issue that can present unique diagnostic and treatment challenges (Fox et al., 2023; Sutherland et al., 2025).

There are somewhere in excess of 100 forms of dementia (Alzheimer’s Disease International, 2026; Wong and Chui, 2022). Alzheimer’s disease (AD) accounts for 60–80% of dementias (Beydoun et al., 2020; Litke et al., 2021) with vascular dementia (VaD) representing 10–20% of dementias (Karamacoska et al., 2023). While these two dementias represent the most common ADRDs, the other three most common subtypes are dementia with Lewy bodies (DLB), frontotemporal dementia (FTD), and mixed dementia (MD). Compounding the complexity of the many types of ADRDs is a change in terminology from “dementia” to “neurocognitive disorder” (NCD) that occurred in the fifth edition of the Diagnostic and Statistical Manual of Disorders (American Psychological Association, 2013). Some advantages of the NCD designation include the ability to classify NCDs as major or mild, the ability to add a descriptive specifier, which is often the dementia subtype involved, and NCD makes room of other diagnoses impacting cognition that are not dementias, such as traumatic brain injury (TBI), for example. Regardless of the pathology, cognitive and later functional decline are hallmarks of ADRDs. Recent literature on advances in earlier dementia testing reveals increased ability to identify (1) previously undetected preclinical and prodromal stages of disease (Liu et al., 2021), (2) noninvasive or minimally invasive blood-based marker, digital phenotyping, eye/retinal markers, magnetoencephalography (MEG) neuroimaging (Alty et al., 2022; Bellomo et al., 2024; Duggan et al., 2025; Sunde et al., 2026), and (3) biomarker-informed cohort screening and preventative trial research advances(Alty et al., 2022; Duggan et al., 2025; Hickman et al., 2023).

### Progressive nature of ADRD

**Figure 1:**
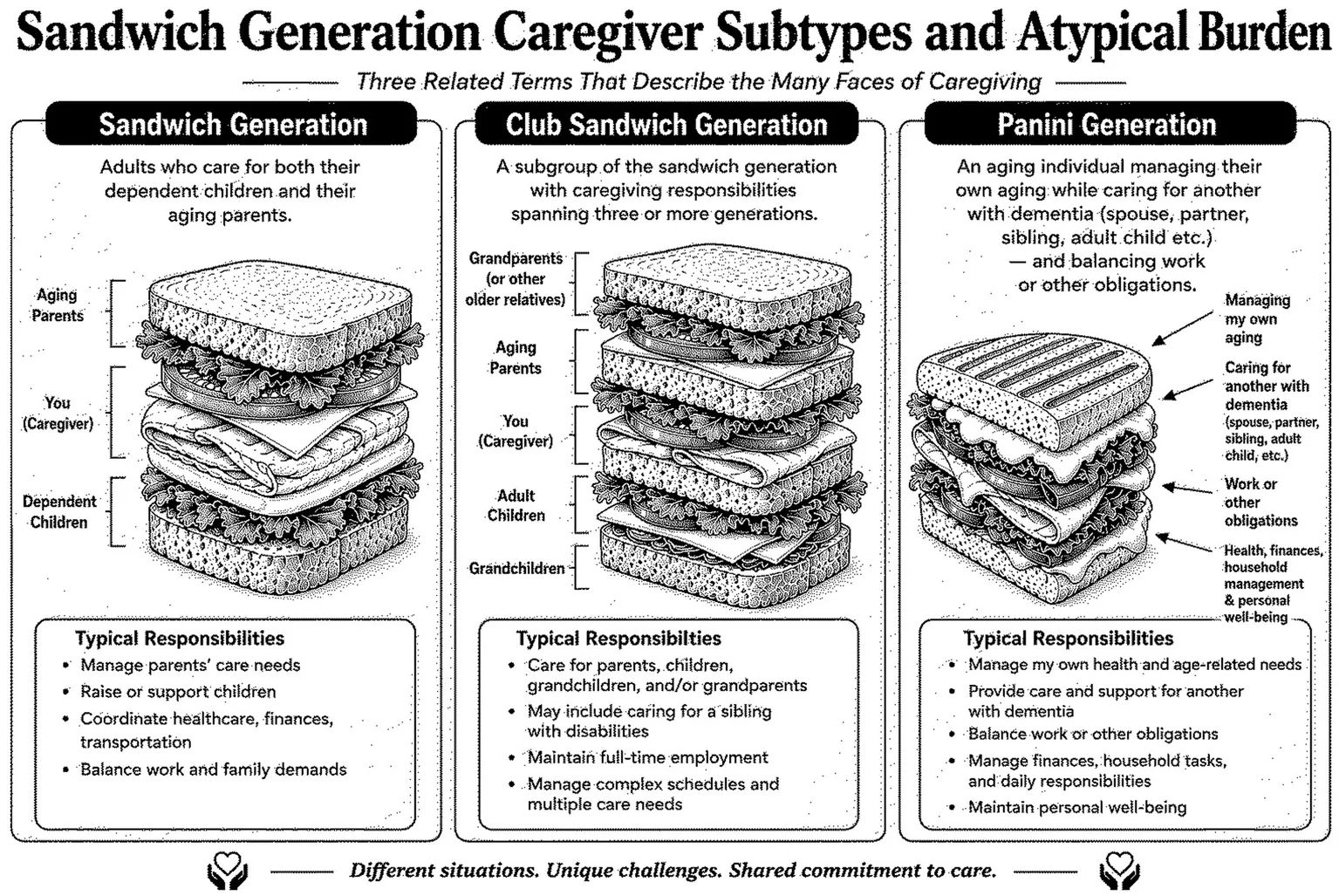
Sandwich Generation Caregiver Subtypes and Atypical Burden was created with the assistance of ChatGPT (OpenAI, GPT-5.5) to illustrate our understanding of Abramson’s (2015) overview of the different subtypes of sandwich generation caregivers. The prompts used for this image are provided in Supplementary material.

ADRDs are a significant group of cognitive disorders marked by declines in memory, executive function and reasoning, orientation, language, attention and concentration, and visuospatial abilities (Yan et al., 2024; Yang, 2025). Behavioral symptoms are associated with NCDs across etiologies (Volicer, 2018) and can result in rapid disease progression and high caregiver burden (Gitlin et al., 2018). A recent meta-analysis revealed that life expectancy after an ADRD diagnosis can vary greatly by age at diagnosis, gender, and type of NCD ranging from 2.2 to 13 years (Brück et al., 2025). ADRD diagnoses are linked to declines in functional ability and independence that result in the need for dependent caregiving assistance (Kawaharada et al., 2019). Much of the caregiving is provided by family members (e.g., spouses and adult children) who may experience financial and emotional burdens as well as health-related difficulties because of their informal caregiver role. With earlier diagnostic capabilities and growing recognition of atypical variants of ADRDs, unique caregiver roles and experiences may arise. Literature documents caregiver burdens in general, but current research and practice do not sufficiently address the experience of all caregivers such as sandwich generation, club sandwich generation, and panini sandwich generation caregivers (Figure 1).

### Atypical caregiver roles and unique challenges

Complex atypical caregiver roles may include role reversal or multiple roles. Some examples of these shifting caregiver dynamics include children becoming caregivers, or a parent serving as a caregiver for their adult child with YOD. MCI before the age of 65 is less likely to be correctly attributed to ADRD and the emotional toll of caring for individuals with YOD is heightened by ambiguity in the earlier stages of the disease due to the resulting misdiagnoses and delay in accurate diagnosis (Kilty et al., 2019). An example of multiple roles would be if someone with mild cognitive impairment related to ADRD served as a caregiver for a loved one who has progressed into dementia. Complex atypical caregiver roles are accompanied by unique and often poorly addressed burdens.

### Caregivers with multiple roles

The Alzheimer’s Association (2025a) cited that over half of ADRD caregivers are taking care of their parents or in-laws, with a quarter of caregivers being part of the sandwich generation. This latter group must balance caring for aging parents with raising their children and in some cases their grandchildren. In a recent study in the United States (Fenstermacher et al., 2026) researchers found that burnout was higher for parent-only relative to child-only caregivers, but the risk was even higher for sandwiched caregivers. In contrast, Shi et al. (2024) found better cognitive health for child-only and the sandwich generation caregivers compared to parent-only caregivers in a large sample (*n* = 8,065) from China. Studies such as these suggest that caring for progeny is a more positive experience than being a caregiver for parents or grandparents (Lai et al., 2023; Page et al., 2018). Although not always true, parenting may be a more welcomed and intrinsically rewarding experience than caring for parents, which is complicated by emotional challenges related to role reversals and anticipatory grief. The authors interpret the discrepant findings between the Fenstermacher and Shi research groups may be attributable to differences in individualistic and collective ideological frameworks impacting caring for older adults. Children taking care of their parents is not uncommon, but there is another subpopulation that is overlooked in the literature.

### Role reversal for younger caregivers

People caring for their parents with YOD may be younger than those from the sandwich caregiver population. The average age of adult children serving as informal caregivers for their parents in Fenstermacher et al. (2026) study was 40.21 years (*R* = 20–70). Individuals diagnosed with ADRDs earlier in life are likely to have children who are closer to the lower limit of that age range. Younger disease onset can result in the loss of income to the household due to the need to quit working well before being eligible for retirement benefits. With YOD, the application process for disability benefits is daunting and criteria for service eligibility may unfairly overly consider advanced age-related to eligibility (Novek and Menec, 2022). Moreover, young adults are still learning how to integrate self-care and therefore may face additional difficulties in becoming a caregiver for a parent living with YOD and navigating these complex issues. Kilty and colleagues also found that young adult caregivers are unprepared for role changes that accompany the early onset of ADRD. Minor children may be tasked with caregiving duties for parents with YOD. Patrick et al. (2023) offered one of the only studies that addressed grandchildren as informal family caregivers, and furthermore, they included children as young as 14 in their sample, with those caring for grandparents with NCDs reporting higher levels of depression. This is significant given the evidence that depression itself is a risk factor for later NCDs (Pannu and Goyal, 2024). Whether the caregiver is a child or a grandchild, young adults and minors involved in providing informal care for someone with an ADRD faces unique hardships and burden.

### Parents as caregivers

A second complex caregiver role that may arise in the context of YOD, is when a parent cares for their adult child who is diagnosed with ADRD. Literature on these caregiver/recipient dyadic relationships is limited, but there is some emerging research on the topic. Rokach and Gershfeld-Litvin (2026) highlighted the unique challenges that this population faces in a small interview study with caregivers recruited from Israel. Consistent with findings from Kilty et al. (2019), caregivers reported a long diagnostic period and ambiguity in understanding symptoms. The authors’ reports on the parent–child relationship was uniquely within the context of the disease. Parental commitment to the child remained high but with recognition of the required sacrifices of becoming a “lifelong parent” to their adult child (p. 270). Sandwiched caregivers who are older have been referred to as club sandwich carers (as cited in Abramson, 2015). Abramson coined the term “panini sandwich caregivers” for older adults who are stuck between their own age-related declines and caring for a family member. Along with spousal and sibling caregivers to older adults with ADRDs, parents caring for their adult children fall into this category.

### Caregivers with ADRD

One final complex caregiver-recipient dyad is one in which the caregiver is in the early stages of neurodegeneration or develops MCI while serving as a caregiver for a loved one with ADRD. The inability of caregivers to focus on their own health promotion and well-being may accelerate caregiver exhaustion or lead to exacerbation of serious illness and subsequent incapacity among caregivers themselves. A recent analysis by the Public Health Center of Excellence on Dementia Caregiving (Alzheimer’s Association, 2025a) found that caregivers of individuals with NCD frequently had multiple modifiable risk factors associated with impaired cognitive functioning and increased dementia risk. Jeffers et al. (2021) conducted a large, multi-state analysis of unpaid caregivers ≥45 years of age from 2015 to 2019 identifying absolute prevalence in caregiver SSD. Caregivers’ self-reported worsening confusion and memory loss or subjective self-decline (SSD) occurred at a 2.4% higher rate than in non-caregivers. This difference further underscores that potential negative impacts on caregiver cognition may affect their ability to navigate complex cognitive tasks such as giving medications, providing financial management (Jeffers et al., 2021) and communicating with health professionals which could be profound, potentially destabilizing the care of both the caregiver and the individual with NCD. Although not well elucidated in the literature, it is also important to consider that caregivers with their own ADRD diagnosis have a window into their future through the observation of the primary care recipient’s progressive decline. This is bound to take an unusual emotional toll on the caregiver and other family members.

## Discussion and conclusion

With numerous variants of neurocognitive degenerative disorders and the Alzheimer’s Association (2025b) report that the majority of support comes in the form of informal caregivers, there is no question that consideration of caregiver experiences needs continued exploration. In this perspective piece, we have examined some unique and complex atypical caregiver roles. Specific to children serving as caregivers in the context of ADRD, we differentiated younger caregivers from those of the sandwich generation. We also highlighted the emerging literature on parents caring for their adult children diagnosed with ADRD. Finally, we addressed caregiver and care recipient dyads who both have an ADRD.

Emerging from our review of the literature on children serving as informal caregivers was the suggestion that support systems in place for aged individuals with ADRD are not well suited for those caring for someone with YOD (Kilty et al., 2019). This is important because research (Juntunen et al., 2018) revealed that high-quality support and lower levels of cognitive challenges of the ADRD care recipient were associated with lower caregiver burden. Although not broken out across caregiver groups, participants in another study (Helvik et al., 2024) also described that challenging behaviors experienced by individuals diagnosed with a YOD and who had unmet support needs contributed to caregiver burden. A common coping mechanism for younger caregivers is detachment; both cognitively and emotionally, as well as by creating physical distance. Younger caregivers also tended to find strong social support networks with others in similar situations (Johannessen et al., 2016). Tang et al. (2024) revealed similar challenges in their research, including adapting to unexpected roles, emotional difficulties related to losing a parent to YOD, and the desire for support. Consistent with this study, Wiggins et al. (2023) discussed that children of those with YOD feel responsibility for the well-being of the unaffected parent. This systematic review also described the emotional toll of the uncertainty surrounding concern about disease heredity, amplifying the risk of young caregivers delaying or changing plans for their own lives. Collectively, the literature highlights that increasing support both in the context of personal relationships as well as decreasing barriers to resource access suitable for families facing YOD is imperative in reducing caregiver burden for child carers.

Parents who care for their adult children with ADRD may be considered part of the panini sandwich generation. Some of the additional support these caregivers provide may include managing finances, driving, and mobility issues associated with physically assisting the care recipient. Moreover, parent carers of children with ADRD have the unique and emotionally complex toll of shifting from a proud parent of an autonomous adult child to the anticipatory grief associated with the loss of a loved one to NCD symptoms. Of course, this is true for all loved ones of individuals diagnosed with ADRDs, but the unexpected nature of being an observer of one’s adult offspring reverting to a dependent, child-like state can be particularly difficult.

With 1 in 9 individuals over the age of 65 and approximately 200,000 younger adults having a diagnosis (Alzheimer’s Association, 2025b), it is important to consider the potential for both a caregiver and a primary care recipient to have ADRD diagnoses simultaneously. This is a concerning possibility given that half of the spousal caregivers are sole providers of informal care during the last years of life (Ornstein et al., 2019). At a minimum, clinicians and hospice staff should consider monitoring not just the patient with ADRDs but should also include caregiver screening for the onset of MCI to help ensure the safety of the dyad. Mutual interconnectedness between caregiver and care recipient wellbeing and adaptation has the potential to affect both persons in the caregiving dyad (Ferraris et al., 2022). Dyadic relationships between dementia carers and care recipients are often fragile and require ongoing evaluation and support throughout the illness trajectory.

Yang et al. (2021) found that shared environments may underlie the risk of cognitive decline in individuals who care for their spouse with NCD symptoms. It is possible that addressing modifiable risk factors could provide caregivers with some protection against developing ADRDs.

This unique and complex caregiver and recipient relationship is likely understudied and addressed given the statistics on prevalence surrounding ADRDs. There are limited longitudinal data tracking resilience experiences of ADRD caregiving subgroups and even less among atypical and complex minority subgroups. While various three-phase dyadic caregiver adaptation and interdependence models have been hypothesized, larger and more rigorous dyadic longitudinal research focused on the resilience processes and which specific interventions sustain resilience over time remain important opportunities within the biomedical and behavioral sciences (Perales-Puchalt et al., 2020; Piiparinen and Whitlatch, 2011).

Sharing caregiving roles across family members can be especially useful for the groups we discuss in this perspective piece. Younger caregivers may be at heightened risk due to incomplete maturation and minimal life experiences. Parents caring for their adult children may be unable to meet the needs of the care recipient due to constraints related to their own aging. Both parent and spousal or sibling carers who are older may be subject to the onset of MCI or even later ADRD symptoms given their age and the risks associated with cognitive decline often associated with caregiver burden. Much like a relay race, hand-offs may need to occur based on caregiver capacity to help minimize caregiver strain and burn out. Unfortunately, many caregivers are solely responsible or bear a disproportionately heavy burden for their care recipients leading to extreme strain. Our final suggestion is that there be more focus on further research combined with policy and practice resources and support for families and caregivers of individuals with younger ADRDs and other complex atypical impairments since our review of the literature suggests that resources for aging populations do not readily transfer to these populations.

Identification of emerging resources for complex atypical caregivers is critical for this underserved population. The Center for Medicare and Medicaid Services (CMS) improved dementia experience GUIDE model is one such resource to consider. This emerging national coordinated care and caregiver support model includes caregiver supports, such as care planning development, 24/7 resource access, caregiver education and training, care coordination and referral, and limited, but notably reimbursable respite support, which is a novel and important service offered in this model (Centers for Medicare and Medicaid Services, 2025). Alzheimer’s Association (2024) offers a selection of caregiver support groups, one of which targets YOD caregivers specifically. Additional resource identification and dissemination remain important aims for wider ranging caregiver support development.

## Data Availability

The original contributions presented in the study are included in the article/Supplementary material, further inquiries can be directed to the corresponding author.
