# Supplementary material for: Complex atypical caregiving: navigating the margins of unique caregiver roles and burdens
Source: Front Psychol. 2026 Aug 24;17:1819388. doi: 10.3389/fpsyg.2026.1819388 (PMC13547481; doi:10.3389/fpsyg.2026.1819388)
Supplement: Supplementary file 1 [file Table_1.docx]

# AI Figure Generation Prompt (Archived for Reproducibility)

**Figure Title:** Sandwich Generation Caregiver Subtypes and Atypical Burden

## Prompt

Create a publication-quality black-and-white line-art infographic for an academic journal with a white background, black outlines, serif title font, and a symmetrical three-column layout. Remove decorative color, shading, logos, icons, and the Key Distinctions section.

Title: Sandwich Generation Caregiver Subtypes and Atypical Burden
Subtitle: Three Related Caregiving Typologies Across the Adult Lifespan

Panel 1 – Sandwich Generation
Definition: Adults simultaneously caring for dependent children and aging parents.
Illustrate a sandwich labeled Aging Parents, You (Caregiver), and Dependent Children.
Typical Responsibilities:
• Manage parents' care needs
• Raise or support children
• Coordinate healthcare, finances, and transportation
• Balance work and family demands

Panel 2 – Club Sandwich Generation
Definition: A subgroup of the Sandwich Generation providing care across three or more generations.
Illustrate a club sandwich labeled Grandparents (or other older relatives), Aging Parents, You (Caregiver), Adult Children, and Grandchildren.
Typical Responsibilities:
• Care for parents, children, grandchildren, and/or grandparents
• May include caregiving for a sibling with disabilities
• Maintain employment
• Coordinate multiple caregiving responsibilities

Panel 3 – Panini Generation
Definition: An aging individual managing their own aging while caring for another person with dementia (e.g., spouse, partner, sibling, adult child) while balancing work or other obligations.
Illustrate a grilled panini with callouts:
• Managing my own aging
• Caring for another with dementia (spouse, partner, sibling, adult child, etc.)
• Work or other obligations
• Health, finances, household management, and personal well-being
Typical Responsibilities:
• Manage my own health and age-related needs
• Provide care and support for another with dementia
• Balance work or other obligations
• Manage finances, household tasks, and daily responsibilities
• Maintain personal well-being

Footer:
“Different situations. Unique challenges. Shared commitment to care.”

Design requirements:
• Monochrome only
• High-contrast line art
• Journal-ready typography
• Minimalist academic style
• Suitable for SVG/EPS or 1200 dpi TIFF output.

## Suggested AI Disclosure

Figure concept and initial artwork were generated using OpenAI image-generation tools from author-developed prompts. The authors subsequently reviewed and revised the terminology, layout, and scientific content for accuracy and consistency with the manuscript.
